# Impact of Internet Use on Elderly Health: Empirical Study Based on Chinese General Social Survey (CGSS) Data

**DOI:** 10.3390/healthcare8040482

**Published:** 2020-11-12

**Authors:** Jing Wang, Changyong Liang, Keqing Li

**Affiliations:** School of Management, Hefei University of Technology, Hefei 230009, China; jingwhfut@gmail.com (J.W.); lkqing1995@mail.hfut.edu.cn (K.L.)

**Keywords:** internet use, elderly, physical health, mental health, self-rated health

## Abstract

In the current era, the rapid spread of Internet technology has combined with various traditional industries; this provides new research perspectives and solutions for current problems, such as those in the elderly care industry. Elderly health is an important social problem in various countries, and governments have turned to the internet for new methods and better solutions. However, internet-use behavior has a certain influence on the elderly’s health status. This study investigates the effects of internet use on the elderly’s physical health, mental health, and self-rated health, along with the moderating role of individual cognitive ability in the above relationship. This study uses data from the Chinese General Social Survey (CGSS) in 2012 and 2015 as samples for analysis via the hierarchical regression method. The sample is from China and had 2821 and 3185 valid respondents in 2012 and 2015, respectively. Results show that internet use significantly affects the physical and mental health of the elderly and does not significantly affect self-rated health. In addition, individual cognitive ability plays a negative moderating role between internet use and physical and mental health. Finally, on the basis of results analysis and discussion, this study provides new recommendations to achieve targeted health improvements.

## 1. Introduction

Statistics have shown that the number of China’s elderly people (over 60 years old) exceeded that of people younger than 15 for the first time in 2018 [1]. In 2019, China had 253 million people aged 60 and over, which accounted for 18.1% of the total population, while those over 65 years accounted for 12.6% [2]. Population aging mainly brings two problems: economic problems such as pensions and other expenditures for elderly services, where the solution depends on the development of China’s economy and the improvement of social security, and elderly health problems. Health problems are not only related to individual survival and development but also to the country’s future and fate. Thus, health problems are important issues in developed and developing countries [3]. Ultimately, finding a reasonable strategy to improve elderly health is significant.

Similarly, the internet is integrated into various traditional industries, such as elderly care [4], government services [5], education [6], and finance [7]. According to “The 45th China Statistical Report on Internet Development”, released by the China Internet Network Information Center [8], internet users over 50 years old reached 16.9% of the population as of March 2020. Internet use has penetrated the elderly group and has gradually changed their daily lifestyles to a certain extent [9]. For example, an increasing number of activities, such as socializing, shopping, and elderly entertainment, are transitioning from offline to online. As an important platform and tool, the internet increases people’s contact with the outside world. The internet can provide people with more social contact, social support, and positive attitude toward life [10,11]. The specific relationship between internet use and elderly health deserves further study. Thus, such a topic has been increasingly explored to determine effective programs to improve the latter.

Instead of focusing on the relationship between internet use and only one single dimension of health, this research includes three dimensions and a moderating variable named individual cognitive ability, which is absent in other research. This study uses samples from the Chinese General Social Survey (CGSS) in 2012 and 2015, which included 2821 and 3185 valid respondents in 2012 and in 2015, respectively. Data from the two years can show the changing impact of internet use on multidimensional elderly health.

The remainder of this study is organized as follows: Section 2 introduces related works; Section 3 presents the method, including the study design, measures, and analysis method; Section 4 details the results; Section 5 presents a discussion; finally, Section 6 summarizes and provides the limitations and implications of this study.

## 2. Related Works

As people age, their physiological and socioeconomic status and social role change. Their health condition deteriorates, and their prevalence and morbidity increase significantly. Therefore, the elderly are the largest “health-vulnerable” group in China [12]. We divided elderly health status into self-rated health, physical health, and mental health. Self-rated health refers to the subjective and personal perception of individuals about their overall health [13,14], whereas physical health refers to a state in which an individual is free from disease at the physical level [15]; mental health refers to an individual’s good mental, emotional, and conscious states [15].

Significant factors influencing elderly health are gender, age, marital status, and socioeconomic status [16,17]. For example, Wang proposed that elderly females suffer poorer health than elderly males in China [18]. Angelini believes that elderly health declines with age to a certain extent [19]. Manaf believes that the elderly who are single (unmarried, divorced, or widowed) are more likely to suffer from depression than those who are married because, in later life, married life and having a life partner can provide the elderly with a sense of security and, thus, better health [20]. Socioeconomic status includes education level and household income [21]. Higher socioeconomic status also significantly affects elderly health [22]. In contrast, low socioeconomic status negatively impacts health, is more likely to be in an adverse environment, and generates negative emotions or potential stress [23]. Wang argued that the elderly with higher household incomes have better living conditions and medical services [24]. Cutler argued that well-educated people are less likely to smoke, less likely to be obese, and less likely to drink; this leads to better elderly health status [25]. Ultimately, many factors affect elderly health status, such as age, gender, marital status, and socioeconomic status. Thus, we add these factors to the model as control variables.

Recent studies have shown that internet use also affects the elderly person’s self-rated health, physical health, and mental health. In self-rated health, Enrique showed that Internet use can significantly improve the elderly’s self-rated health. For example, compared with non-internet users, the self-rated health of internet users is significantly higher from closer contact with the outside world [26]. In physical health, Wang found that internet use has a significant improvement effect on elderly physical health by using data from the CGSS in 2013 [27]. For example, internet usage can facilitate the access and use of relevant information and services, particularly for medical problems [28,29]. The elderly with high blood pressure and heart disease can significantly reduce their incidences by using the internet to browse and gain knowledge about prevention and healthcare [30]. For mental health, David believes that a direct relationship exists between internet use and elderly mental health [31]. For example, the more frequently the elderly use the internet, the less lonely they feel in later life; their life satisfaction and mental health improved [32]. Lelkes uncovered that the elderly can effectively improve their subjective wellbeing by using the internet to maintain close contact with society [33]. Fundamentally, the influence of internet use behavior on elderly health status is multifaceted. Through internet use, the elderly can obtain useful information and health knowledge, reduce loneliness, expand social contacts, and maintain close contact with friends and family, ultimately improving their health status. On the basis of the above discussion, we assume that

**Hypothesis** **1.** Internet use significantly impacts elderly self-rated health.

**Hypothesis** **2.** Internet use significantly impacts elderly physical health.

**Hypothesis** **3.** Internet use significantly impacts elderly mental health.

Individual cognitive ability refers to the ability of the human brain to process, store, and extract information [34]; it is also one of the important factors that determine whether the elderly can live independently [35]. With an increase in age, the incidence of cognitive impairment in the elderly escalates sharply [36], and manifestations such as slower reaction, memory decline, and decline in anti-interference ability become apparent [37,38]. The decline of cognitive ability can also directly affect the daily life of the elderly, such as cooking, financial management, medical treatment, and external activities [39]. Individual cognitive ability and health level are the two most important aspects of aging. For example, the cognitive decline in elderly people may develop into mild cognitive impairment or Alzheimer’s disease [34]. Conversely, higher cognitive ability and good cognitive literacy, which enable people to gain an understanding of and execute complex treatment plans and better disease self-management capabilities, can directly increase health levels [40]. In addition, cognitive ability significantly and positively impacts internet use because internet use is a highly cognitive activity that integrates the processes of memory, processing, reasoning, and learning; older people with higher cognitive scores are more likely to master internet technology [41].

Ultimately, in the context of the rapid development of the internet, a certain relationship exists among individual cognitive ability, internet use, and elderly health. Concurrently, we think that elderly health includes self-rated health, physical health, and mental health. Therefore, we explore individual cognitive ability as a moderating variable and study the relationships between internet use and self-rated health, physical health, and mental health to explore the role of individual cognitive ability on internet use and elderly health. On this basis, we assume that

**Hypothesis** **4.** Individual cognitive ability plays a moderating role between internet use and elderly self-rated health.

**Hypothesis** **5.** Individual cognitive ability plays a moderating role between internet use and elderly physical health.

**Hypothesis** **6.** Individual cognitive ability plays a moderating role between internet use and elderly mental health.

**Figure 1:**
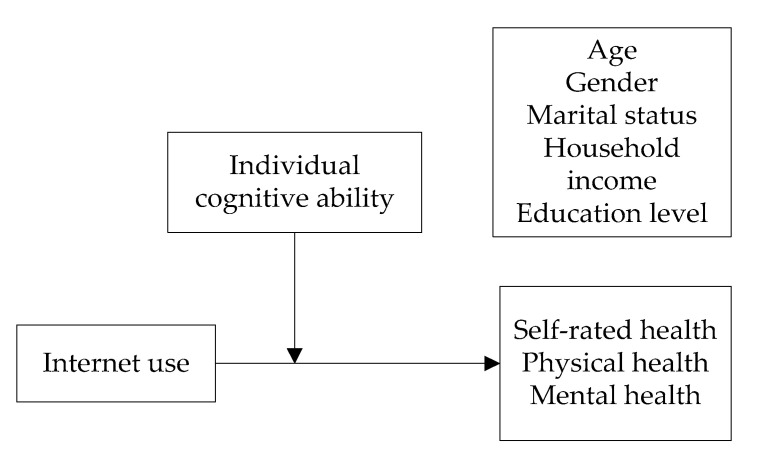
Research model.

Previous research on the relationship between internet use and elderly health has mainly focused on a single dimension, such as mental health or self-rated health [42,43,44]. Integrated research on self-rated health, physical health, and mental health of the elderly remains lacking. Thus, this study aims to explore the relationship between these three dimensions of health and internet use in order to provide guidance and help improve elderly health. Meanwhile, this study selects samples from different years to study the changes in the impact of internet use on health over three years. To further understand the impact of internet use on elderly health and find ways to improve elderly health, this study selects individual cognitive ability as the moderating variable. Figure 1 shows the theoretical model.

## 3. Methods

### 3.1. Study Design

This study uses data from the Chinese General Social Survey (CGSS) for two main reasons. First, CGSS has variables related to individual internet use and contains specific information such as personal and family characteristics and socioeconomic and health status. Second, CGSS is the earliest national, comprehensive, and continuous academic survey project in China; it adopts multistage stratified sampling, which is currently recognized as representative data with scientific research value in academia [45,46]. The research object is the elderly population over 60 years old. The samples comprised 11,765 and 10,968 respondents in 2012 and 2015, respectively. Screening and eliminating samples with incomplete variables yielded 2821 and 3185 valid respondents in 2012 and 2015, respectively.

### 3.2. Measures

#### 3.2.1. Internet Use (Independent Variable)

In the internet-use selection questionnaire, the main predictor is, “In the past year, what was your use of the internet (including mobile internet)?”. Based on the respondents’ answer, the rating in terms of the frequency on a 5-point scale ranged from “never” (=1), “rarely” (=2), “sometimes” (=3), “often” (=4), and “very frequently” (=5).

#### 3.2.2. Health Status (Dependent Variable)

This study assesses elderly health status in three aspects, namely, self-rated health, physical health, and mental health. In the self-rated health selection questionnaire, the main predictor is, “What do you think of your current health?”. Respondents’ answers ranged from “very unhealthy” (=1), “relatively unhealthy” (=2), “average” (=3), “relatively healthy” (=4), and “very healthy” (=5). In the physical health selection questionnaire, the main predictor is, “In the past four weeks, how often has your work or other daily activities been affected because of health issues?”. In the mental health selection questionnaire, the main predictor is, “In the past four weeks, how often have you felt depressed?” For physical health and mental health, the rating regarding the frequency on a 5-point scale ranged from “always” (=1), “often” (=2), “sometimes” (=3), “rarely” (=4), and “never” (=5).

#### 3.2.3. Individual Cognitive Ability (Moderating Variable)

Individual cognitive ability includes many factors, such as mathematical, spatial, and language ability [47]. Among them, language ability is the key to cognitive ability because communication is a core function that can accurately express one’s ideas; language ability involves core language, language fluency, and language content [48]. In the CGSS questionnaire, this study used four questions that indicate individual cognitive ability: (a) How good is your ability to understand Mandarin?, (b) How good is your ability to speak Mandarin?, (c) How good is your ability to understand English?, and (d) How good is your ability to speak English? According to the level of language mastery, the ratings ranged from “completely incomprehensible (cannot speak at all)” (=1), “relatively poor” (=2), “average” (=3), “better” (=4), and “very good” (=5). This study obtains individual cognitive ability by multiplying these four questions by 1/4 and then adding them together.

#### 3.2.4. Sociodemographic Characteristics (Control Variable)

Five variables are included: age, gender, marital status, household income, and education level. Gender is coded as 0 for females and 1 for males. Marital status is measured with a dummy variable—1 for married and 0 for unmarried. Educational levels are expressed as “no education” (=1), “primary school” (=2), “middle school” (=3), “high school” (=4), “junior college” (=5), “undergraduate” (=6), and “graduate and above” (=7).

### 3.3. Hierarchical Regression Analysis

The experimental platform of this study is SPSS 25.0(Beijing Cloud-wing Data Information Technology Co.,Ltd, Beijing, China). This study aims to explore how internet use affects self-rated health, physical health, and mental health in the elderly via two steps. First, descriptive statistics and analysis are applied to the valid data from 2012 and 2015. Second, hierarchical regression analysis of the two years of data is used to study the effects of internet use on self-rated health, physical health, and mental health of the elderly and, subsequently, with the moderating effect of individual cognitive ability.

## 4. Results

### 4.1. Descriptive Statistics

This study uses descriptive statistics to analyze the mean and standard deviation of measurement variables. Table 1 shows the statistical results. The sample shows that from 2012 and 2015, the average internet use is 1.22 and 1.35, respectively, indicating a rate increase of 0.13. According to Table 1, the respective mean values of the two years are as follows: elderly self-rated health is 3.01 and 3.18, elderly physical health is 3.42 and 3.45, and elderly mental health is 3.73 and 3.72. The elderly health status in China has improved to a certain extent but remains at a moderate level. The mean of individual cognitive ability is 2.06 and 2.09, which also improved.

### 4.2. Hierarchical Regression Analysis Results

In this study, the data of 2012 and 2015 are analyzed using hierarchical regression. Model 1 includes all the control variables, Model 2 adds individual cognitive ability to Model 1, and Model 3 adds internet use to Model 2. Table 2 shows the influence of internet use on elderly self-rated health. Model 1 shows that age, gender, household income, and education level correlate with elderly self-rated health. In contrast, marital status is not significant. Model 2 shows that individual cognitive ability significantly and positively affects self-rated health. The coefficient of correlation in 2015 (coefficient = 0.270, *p* < 0.001) is greater than that in 2012 (coefficient = 0.224, *p* < 0.001). Model 3 shows that although internet use is positively correlated with self-rated health, the relationship is not significant, and thus Hypothesis 1 is not supported.

Table 3 shows the influence of internet use on elderly physical health. According to Model 1, the 2012 data show a correlation between age, gender, education level, and physical health. In the 2015 results, household income is also correlated with physical health. This suggests that the elderly with higher household income have better physical health. Model 2 shows that individual cognitive ability is positively correlated with physical health. The coefficient of correlation in 2015 (coefficient = 0.331, *p* < 0.001) is greater than that in 2012 (coefficient = 0.243, *p* < 0.001). Model 3 shows that internet use is significantly and positively correlated with physical health, and thus, Hypothesis 2 is supported. The coefficient of correlation in 2015 (coefficient = 0.064, *p* < 0.01) is smaller than that in 2012 (coefficient = 0.106, *p* < 0.01).

Table 4 shows the influence of internet use on elderly mental health. The results of Model 1 show a correlation of marital status, household income, and education level with mental health in the 2012 data. In 2015, gender and education level are correlated with mental health. The possible reason is related to the unequal proportion of male and female respondents. Model 2 shows that individual cognitive ability is positively correlated with mental health. The coefficient of correlation in 2015 (coefficient = 0.250, *p* < 0.001) is smaller than that in 2012 (coefficient = 0.329, *p* < 0.01). Model 3 shows that internet use is significantly and positively correlated with mental health, and thus, Hypothesis 3 is supported. The coefficient of correlation in 2015 (coefficient = 0.057, *p* < 0.01) is smaller than that in 2012 (coefficient = 0.083, *p* < 0.01).

Table 5 shows the results of the linear regression model of the interactive effects of self-rated health, physical health, and mental health. The two years of data show no moderating role of individual cognitive ability between Internet use and self-rated health, and thus, Hypothesis 4 is not supported. Individual cognitive ability has a negative moderating role between internet use and physical health, and thus, Hypothesis 5 is supported. Individual cognitive ability has a negative moderating role between internet use and mental health, which supports Hypothesis 6. Compared with the elderly with high individual cognitive abilities, those with low individual cognitive abilities are more likely to improve their physical and mental health after using the internet.

## 5. Discussion

This study focuses on the impact of internet use on elderly health. Three dimensions of health exist: self-rated health, physical health, and mental health. Previous research has used data from only one year to explore the impact of internet use on a single dimension of health, such as physical health or mental health. In comparison with those studies, a variation trend can be obtained by observing the difference in results over a three-year interval in this study. In addition, this study focuses on health status from more than one dimension.

The following revelations can be drawn from the results of the experiment. From the comparison of the 2012 and 2015 results, internet use does not significantly affect self-rated health; thus, Hypothesis 1 is not supported.

Internet use significantly and positively affects physical health; thus, Hypothesis 2 is supported. It indicates that elderly physical health can be improved by increasing internet use behavior, which is consistent with previous findings on single-level health and proves the value of the present study [49].

Internet use significantly and positively affects mental health; thus, Hypothesis 3 is supported. It indicates that the more the elderly use the internet, the better their mental health will be, which is also consistent with previous findings on single-level health [43,44].

Individual cognitive ability does not play a moderating role between internet use and elderly self-rated health; thus, Hypothesis 4 is not supported.

Individual cognitive ability plays a moderating role between internet use and elderly physical health; thus, Hypothesis 5 is supported. Contrary to expectations, the moderating effect is negative; that is, the elderly with higher cognitive abilities would benefit less when improving physical health via the same internet-use behavior. An effect called diminishing returns exists, which means that when improving elderly physical health via internet use, the level of improvement of physical health decreases with increasing internet use.

The moderating role of individual cognitive ability between internet use and elderly mental health is also verified; thus, Hypothesis 6 is also supported. The elderly with higher cognitive abilities would benefit less when improving mental health via the same internet use behavior, and the effect called diminishing returns also exists; that is, when improving elderly mental health via internet use, the level of improvement of mental health decreases with increasing internet use. The above results can be used to help determine ways to improve elderly health.

## 6. Conclusions

The internet has become increasingly popular in the daily lives of the elderly in China. To better explore the relationship between internet use and the self-rated health, physical health, and mental health of the elderly and the correlation and the moderating role of individual cognitive ability, this study selected national data from 2012 and 2015 and added individual cognitive ability to the model as a moderating variable. The results show that for the elderly with stronger individual cognitive abilities, internet use behavior minimally affects physical health and mental health. This finding suggests that the effects of internet use on elderly health are related to their own cognitive abilities. On this basis, suggestions for improving elderly health status are further put forward.

This finding is of considerable value to communities and public service organizations. Staff in communities or public service organizations need to keep investing extensive efforts to promote internet-use behavior in the elderly and to be more targeted in their work to maximize the benefits of health improvements in the elderly. They should also pay more targeted attention to the elderly with weak individual cognitive ability because the improvement of their health caused by internet use will be more obvious.

This study has several limitations. The internet use behavior of the elderly covers different aspects, such as social contact, shopping, and travel. However, this study does not specify the internet usage of the elderly, which requires further data support.

Future research may try to understand how different internet use behaviors (online social networking, online shopping, mobile video, online travel, mobile reading) specifically affect elderly health status. This may assist community employees and public service organizations in identifying targeted health outcomes for the elderly.

## Figures and Tables

**Table 1:** Descriptive statistics.

Variable	2012		2015	
	Mean	Standard Deviation	Mean	Standard Deviation
Internet use	1.22	0.785	1.35	0.934
Self-rated health	3.01	1.053	3.18	1.062
Physical health	3.42	1.217	3.45	1.117
Mental health	3.73	1.038	3.72	0.927
Individual cognitive ability	2.06	0.607	2.09	0.607
Gender	0.56	0.497	0.49	0.500
Age	69.10	7.209	69.39	7.469
Marital status	0.73	0.444	0.74	0.436
Household income	43,072.49	269,983.171	61,361.82	380,656.167
Education level	2.40	1.276	2.39	1.263
*N*	2821	2821	3185	3185

**Table 2:** Results of linear regression models on self-rated health and relevant variables.

Predictor		2012			2015	
	Model 1	Model 2	Model 3	Model 1	Model 2	Model 3
Age	−0.08 * (0.003)	−0.07 * (0.003)	−0.06 * (0.003)	−0.011 *** (0.003)	−0.010 *** (0.003)	−0.010 *** (0.003)
Gender	0.179 *** (0.041)	0.191 *** (0.041)	0.192 *** (0.041)	0.188 *** (0.039)	0.214 *** (0.038)	0.216 *** (0.039)
Marital status	0.05 (0.048)	0.05 (0.048)	0.05 (0.048)	−0.011 (0.045)	−0.018 (0.045)	−0.021 (0.045)
Household income	1.902 × 10^−7^ * (0.000)	1.690 × 10^−7^ * (0.000)	1.700 × 10^−7^ * (0.000)	1.248 × 10^−7^ * (0.000)	1.266 × 10^−7^ ** (0.000)	1.245 × 10^−7^ ** (0.000)
Education level	0.114 *** (0.016)	0.054 ** (0.019)	0.045 * (0.019)	0.069 *** (0.015)	−0.001 (0.018)	−0.009 (0.018)
Individual cognitive ability		0.224 *** (0.039)	0.215 *** (0.039)		0.270 *** (0.036)	0.261 *** (0.036)
Internet use			0.048 (0.027)			0.033 (0.022)
Constant	3.143 *** (0.217)	2.725 *** (0.228)	2.685 *** (0.229)	3.704 *** (0.199)	3.218 *** (0.207)	3.188 *** (0.208)
Adjusted R^2^	0.042	0.053	0.053	0.026	0.043	0.043

Standard errors are in parentheses. * *p* < 0.05, ** *p* < 0.01, *** *p* < 0.001.

**Table 3:** Results of linear regression models on physical health and relevant variables.

Predictor		2012			2015	
	Model 1	Model 2	Model 3	Model 1	Model 2	Model 3
Age	−0.13 *** (0.003)	−0.11 ** (0.003)	−0.11 ** (0.003)	−0.012 *** (0.003)	−0.011 *** (0.003)	−0.010 *** (0.003)
Gender	0.116 * (0.047)	0.129 ** (0.047)	0.131 *** (0.047)	0.171 *** (0.040)	0.203 *** (0.040)	0.207 *** (0.040)
Marital status	0.006 (0.055)	0.005 (0.055)	0.009 (0.055)	−0.045 (0.047)	−0.054 (0.046)	−0.058 (0.046)
Household income	1.299 × 10^−7^ (0.000)	1.069 × 10^−7^ (0.000)	1.092 × 10^−7^ (0.000)	1.468 × 10^−7^ ** (0.000)	1.508 × 10^−7^ ** (0.000)	1.466 × 10^−7^ ** (0.000)
Education level	0.212 *** (0.018)	0.128 *** (0.022)	0.126 *** (0.022)	0.169 *** (0.016)	0.083 ** (0.018)	0.068 *** (0.019)
Individual cognitive ability		0.243 *** (0.044)	0.224 *** (0.044)		0.331 *** (0.037)	0.314 *** (0.038)
Internet use			0.106 ** (0.031)			0.064 ** (0.023)
Constant	3.719 *** (0.248)	3.264 *** (0.260)	3.176 *** (0.261)	3.851 *** (0.205)	3.256 *** (0.214)	3.197 *** (0.214)
Adjusted R^2^	0.067	0.077	0.080	0.060	0.082	0.084

Standard errors are in parentheses. * *p* < 0.05, ** *p* < 0.01, *** *p* < 0.001.

**Table 4:** Results of linear regression models on mental health and relevant variables.

Predictor		2012			2015	
	Model 1	Model 2	Model 3	Model 1	Model 2	Model 3
Age	0.001 (0.003)	0.003 (0.003)	0.004 (0.003)	−0.001 (0.002)	0.001 (0.002)	0.001 *** (0.002)
Gender	0.078 (0.040)	0.095 * (0.039)	0.097 * (0.039)	0.075 * (0.034)	0.099 ** (0.033)	0.102 ** (0.033)
Marital status	0.142 ** (0.047)	0.140 ** (0.046)	0.143 * (0.046)	0.034 (0.039)	0.027 (0.039)	0.023 (0.046)
Household income	1.684 × 10^−7^ * (0.000)	1.372 × 10^−7^ * (0.000)	1.391 × 10^−7^ * (0.000)	5.670 × 10^−8^ (0.000)	5.838 × 10^−8^ (0.000)	5.465 × 10^−8^ (0.000)
Education level	0.198 *** (0.015)	0.329 *** (0.037)	0.095 *** (0.019)	0.121 *** (0.013)	0.057 ** (0.015)	0.043 ** (0.016)
Individual cognitive ability		0.329 *** (0.037)	0.314 *** (0.037)		0.250 *** (0.031)	0.234 *** (0.032)
Internet use			0.083 ** (0.026)			0.057 ** (0.019)
Constant	3.023 *** (0.210)	2.408 *** (0.219)	2.338 *** (0.219)	3.431 *** (0.173)	2.981 *** (0.180)	2.929 *** (0.214)
Adjusted R^2^	0.077	0.102	0.105	0.033	0.052	0.054

Standard errors are in parentheses. * *p* < 0.05, ** *p* < 0.01, *** *p* < 0.001.

**Table 5:** Results of linear regression models on the interaction effects on self-rated, physical, and mental health.

Predictor		2012			2015	
	Self-Rated Health	Physical Health	Mental Health	Self-Rated Health	Physical Health	Mental Health
Age	−0.006 * (0.003)	−0.010 ** (0.003)	0.004 (0.003)	−0.010 *** (0.003)	−0.010 *** (0.003)	0.001 (0.002)
Gender	0.192 *** (0.041)	0.132 ** (0.47)	0.097 * (0.039)	0.214 *** (0.039)	0.204 *** (0.040)	0.100 ** (0.033)
Marital status	0.050 (0.048)	0.006 (0.055)	0.142 ** (0.046)	−0.024 (0.045)	−0.062 (0.046)	0.019 (0.039)
Household income	1.687 × 10^−7^ * (0.000)	1.066 × 10^−7^ (0.000)	1.375 × 10^−7^ * (0.000)	1.257 × 10^−7^ ** (0.000)	1.481 × 10^−7^ ** (0.000)	5.616 × 10^−8^ (0.000)
Education level	0.045 * (0.019)	0.126 *** (0.022)	0.095 *** (0.019)	−0.009 (0.018)	0.069 *** (0.019)	0.044 ** (0.016)
Individual cognitive ability	0.220 *** (0.039)	0.234 *** (0.045)	0.320 *** (0.038)	0.267 *** (0.037)	0.322 *** (0.038)	0.242 *** (0.032)
Internet use	0.086 * (0.035)	0.178 *** (0.040)	0.126 *** (0.034)	0.057 * (0.027)	0.096 ** (0.038)	0.089 ** (0.023)
Internet use * Individual cognitive ability	−0.029 (0.017)	−0.056 ** (0.020)	−0.033 * (0.017)	−0.027 (0.017)	−0.036 * (0.17)	−0.036 * (0.015)
Constant	2.634 *** (0.231)	3.077 *** (0.263)	2.280 *** (0.221)	3.150 *** (0.209)	3.147 *** (0.216)	2.879 *** (0.182)
Adjusted R^2^	0.054	0.082	0.106	0.044	0.085	0.055

Standard errors are in the parentheses. * *p* < 0.05, ** *p* < 0.01, *** *p* < 0.001.
